# Supplementary material for: An auto-inhibited state of protein kinase G and implications for selective activation
Source: eLife. 2022 Aug 5;11:e79530. doi: 10.7554/eLife.79530 (PMC9417419; doi:10.7554/eLife.79530)
Supplement: Supplementary file 1. — *Information for the highest resolution shell is shown in parenthesis. †5.0% of the observed intensities were excluded from refinement for cross-validation purposes. [file elife-79530-supp1.docx]

**Supplementary File 1. Data collection and refinement statistics**

| Protein | PKG Iβ 71-686 | PKG Iα 79-212 R177Q |
| --- | --- | --- |
| **Data collection** |  |  |
| Wavelength (Å) | 0.97931 | 0.97931 |
| Space group | *P*2_1_2_1_2_1_ | *P*1 |
| Cell dimensions |  |  |
| *a, b, c* (Å) | 99.8, 112.8, 150.7 | 39.2, 41.9, 48.3 |
| α, β, γ (°) | 90, 90, 90 | 94.7, 113.7, 114.0 |
| Resolution (Å) | 47.7 - 2.41 (2.50 - 2.41)* | 36.54 – 1.26 (1.30 -1.26)* |
| R_merge_ | 0.1115 (4.83) | 0.06 (0.175) |
| I/σI | 20.92 (0.84) | 25.4 (6.56) |
| Completeness (%) | 99.95 (99.86) | 93.6 (89.26) |
| Redundancy | 13.4 (13.3) | 3.7 (2.8) |
| **Refinement** |  |  |
| No. reflections | 66392 (6568) | 62350 (3133) |
| R_work_/R_free_^†^(%) | 19.18/23.80 | 17.76/19.69 |
| No. atoms |  |  |
| Proteins | 9208 | 2034 |
| Ligand | 110 | 0 |
| Water | 87 | 361 |
| B-factors |  |  |
| Overall | 84.27 | 23.17 |
| Protein | 84.53 | 20.91 |
| Ligand | 79.41 |  |
| Water | 63.0 | 35.90 |
| R.m.s. deviations |  |  |
| Bond lengths (Å) | 0.009 | 0.009 |
| Bond angles (°) | 1.10 | 1.18 |
| PDB Code | 7LV3 | 7MBJ |

*Information for the highest resolution shell is shown in parenthesis.

^†^ 5.0% of the observed intensities were excluded from refinement for cross validation purposes.
