# Supplementary material for: An auto-inhibited state of protein kinase G and implications for selective activation
Source: eLife. 2022 Aug 5;11:e79530. doi: 10.7554/eLife.79530 (PMC9417419; doi:10.7554/eLife.79530)
Supplement: Supplementary file 2. [file elife-79530-supp2.docx]

**Supplementary File 2. SAXS Data Collection and Scattering-Derived Parameters for PKG Iβ 71-686**

| **Data collection parameters** |  |
| --- | --- |
| Instrument | SIBYLS beamline |
| Wavelength (Å) | 1.127 |
| q range used (Å^-1^) | 0.01 to 0.39 |
| Exposure time (s) | 3 |
| Concentration (mg/mL) | 3.5 |
| Temperature (°C) | 20°C |
| **Structural parameters** |  |
| I(0) (Å) [from Guinier/P(r)] | 96.27/96.27 |
| R_g_  (Å) [from Guinier/P(r)] | 28.16/28.15 |
| Porod volume estimate (Å^3^) | 100286 |
| D_max_ (Å) | 92.7 |
| MW estimated from SAXS data (kDa) | 68.062 |
| Theoretical MW (kDa) | 69.636 |
| Oligomeric state | Monomer |
| **Softwares employed** |  |
| Primary data reduction and data processing | ScÅtter pipelines and ATSAS suites |
| *Ab initio* analysis | DAMMIF/DAMMIN |
| Computation of model intensities | FOXS |
| Fitting of crystal structure in models | SUPCOMB |
