# Supplementary material for: An auto-inhibited state of protein kinase G and implications for selective activation
Source: eLife. 2022 Aug 5;11:e79530. doi: 10.7554/eLife.79530 (PMC9417419; doi:10.7554/eLife.79530)
Supplement: Supplementary file 3. — Specific interactions between the R- and C-domains. The location of each residue within the complex is listed alongside of each amino acid. Ion pair, hydrogen-bond, and van der Waals interactions are notated as ↔, →, and , respectively. [file elife-79530-supp3.docx]

**Supplementary File 3. Specific interactions between the R and C domains**

**
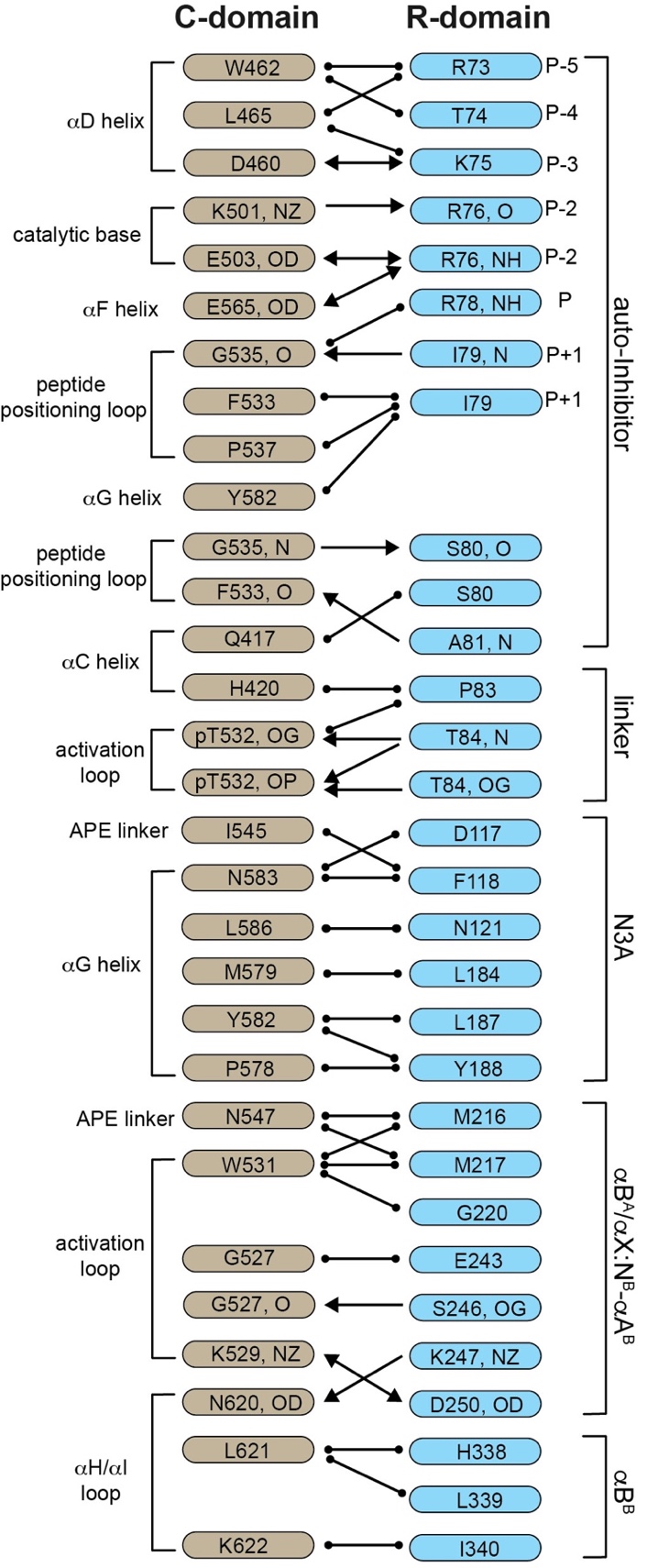
**

Specific interactions between the R and C domains. The location of each residue within the complex is listed alongside of each amino acid. Ion pair, hydrogen-bond, and van der Waals interactions are notated as ↔, →, and 
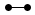
, respectively.
