# Supplementary material for: An auto-inhibited state of protein kinase G and implications for selective activation
Source: eLife. 2022 Aug 5;11:e79530. doi: 10.7554/eLife.79530 (PMC9417419; doi:10.7554/eLife.79530)
Supplement: Supplementary file 4. [file elife-79530-supp4.docx]

**Supplementary File 4. Summary of the MD Simulations Performed for PKG Iβ**

| **Simulated Construct** | **Starting Structure** | **Simulation Length per Replicate (ns)** | **Number of Simulation Replicates Executed** | **Number of CPUs Used** |
| --- | --- | --- | --- | --- |
| Apo Wild-Type CNB-A Domain | Chain A from PDB ID “3OD0” | 579.7 | 1 | 32 |
| cGMP-Bound Wild-Type CNB-A | PDB ID :3OD0 | 591.3 | 1 | 32 |
| Apo RQ-Mutant CNB-A | PKG Iα 79-212 R177Q | 652.3 | 1 | 32 |
| PKG Iβ 71-686 | PKG Iβ 71-686 | 200.0 | 3 | 32 |
